# Psychological capital educational program and its effect on nurse interns’ innovative behavior

**DOI:** 10.1186/s12912-024-02192-5

**Published:** 2024-08-08

**Authors:** Ahmed Hussien Ahmed Kotb, Mona Mostafa Shazly, Hemat Abdel-Azeem Mostafa

**Affiliations:** 1 Department of Nursing Administration, Faculty of Nursing, Fayoum University, Fayoum, Egypt; 2 Department of Nursing Administration, Faculty of Nursing, Ain Shams University, Cairo, Egypt

**Keywords:** Psychological capital, Innovative behavior, Nurse interns, Educational program

## Abstract

**Background:**

Nurse interns’ capacity for innovative behavior is a key component of healthcare settings because of increasing demands and complexities. Thus, it is important to find strategies that promote their innovative behavior. The development of psychological capital (PsyCap) improves positive behaviors and attitudes, such as engagement, motivation, and satisfaction, in the work environment. Therefore, this study aimed to assess the effect of a PsyCap educational program on nurse interns’ innovative behavior.

**Methods:**

A quasi-experimental design was used in this study. This study was executed at Fayoum University hospitals. The study participants comprised all the available nurse interns (*n* = 223) registered in the internship year (2022–2023) in the aforementioned settings. The data were gathered using three instruments, namely, the PsyCap Knowledge Questionnaire, the PsyCap Questionnaire, and the Innovative Behavior Inventory.

**Results:**

The nurse interns’ mean scores regarding total knowledge about PsyCap, total perception of PsyCap, and total perception of innovative behavior significantly improved through the posttest phase (41.27 ± 9.31, 92.22 ± 6.26, 91.31 ± 9.06, respectively) and the follow-up phase (37.83 ± 8.83, 89.96 ± 6.31, 88.89 ± 8.33) in comparison with the pretest phase (14.39 ± 5.83, 69.04 ± 8.13, 60.55 ± 7.15).

**Conclusion:**

The PsyCap educational program was effective and beneficial for improving the nurse interns’ perceptions of innovative behavior. Therefore, PsyCap interventions should be implemented in hospitals through professional development programs and orientation programs.

## Background

Healthcare facilities are obliged to serve in a constantly changing and unexpected climate, which raises risks and creates competitive hurdles. This has resulted from the increase in medical knowledge and the frequent advancement of medical science and technology. Thus, healthcare settings should depend on nurses who perform nursing practices innovatively in order to maintain a competitive edge [1]. In this context, it’s necessary to identify strategies that develop nurse interns’ innovative behavior. One of these strategies is the development of psychological capital (PsyCap) [2].

Nurse interns confront complex challenges and various stressors that impede their ability to apply professional skills and limit their innovative behavior [3]. Additionally, they experience negative emotions and feelings, such as anxiety, depression, and burnout, during the internship year [4]. Nurse interns with high PsyCap cope with difficulties and achieve their desired goals. Additionally, they respond positively to work challenges [5]. Thus, it’s important to improve nurse interns’ PsyCap to perform their duties innovatively [6].

PsyCap is derived from positive organizational behavior (POB). POB focuses on the application and study of positively oriented human resource strengths and psychological capacities that can be measured, developed, and effectively managed for performance improvement in today’s workplace [7]. PsyCap is defined as a positive appraisal of circumstances and the probability of success based on motivated effort and perseverance [8]. Additionally, PsyCap refers to an individual’s preferable psychological condition of development, which consists of four essential constructs: self-efficacy, hope, resilience, and optimism [9].

Self-efficacy points out nurses’ belief in their ability to perform job demands with high standards. Nurses with high self-efficacy typically have greater confidence in handling work responsibilities and finding long-term solutions [10]. Hope refers to the desire to achieve goals as well as the capacity to identify and pursue paths that accomplish them. Hopeful nurses provide numerous approaches to resolve their problems, and they continuously attempt to achieve their aims [6]. Additionally, resilience refers to the ability to bear and recover from different setbacks and diversities. Resilient nurses successfully manage high work demands and have the ability to come back to their normal condition [9]. Optimism refers to the tendency to maintain positive expectations for the future. When optimists are faced with uncertain situations at work, they expect good things to happen, so they will continue and increase their effort**s** [11].

Nurses with high perceptions of PsyCap have a positive vision related to their work. PsyCap enables nurses to cope better in the workplace [12]. PsyCap is related to a number of positive attitudes and behaviors, such as job performance, organizational citizenship behavior, work engagement, and job satisfaction [8, 11]. Additionally, PsyCap has a negative correlation with job turnover, work absenteeism, and stress in the workplace [13]. As well, PsyCap is related to safety performance and the prevention of occupational accidents and injuries in the workplace [14].

PsyCap is susceptible to alteration and development because it is described as a “state-like” variable instead of a “trait-like.” Therefore, PsyCap can be developed through different approaches and educational programs. The most well-known model is the PsyCap intervention (PCI). A PCI is simple training that contains a two-hour session for improving PsyCap through various exercises. These exercises focus on raising individuals’ perceptions of self-efficacy, hope, resilience, and optimism [15]. Another model for increasing PsyCap levels is positive psychology intervention (PPI). PPIs are a set of long-term approaches and exercises that primarily aim at increasing positive feelings, positive cognitions or positive behavior, and well-being, which in turn improve PsyCap [16]. These approaches include the positive focus approach or gratitude, stress management approach, problem solving approach, personal resources interventions, rational-emotive therapy (RET) approach, PERMA model that stands for positive emotion, engagement, relationships, meaning, and accomplishment, job crafting intervention, and career development approach [17].

Numerous studies have shown that components of PsyCap act as antecedents and resources for employees’ innovative behavior [6]. Individuals with high levels of self-efficacy tend to be more confident and motivated in the face of their daily duties, so they seek to define and apply innovative solutions in their companies. Additionally, individuals with high levels of optimism have positive expectations about the results, so they become more creative and willing to provide new and effective solutions [2]. Moreover, individuals who possess hope explore new paths to achieve their goals with persistence. They also recall positive emotions. These allow them to establish an environment that supports innovative attitudes. Lastly, highly resilient individuals tend to be more adaptable and flexible when placed in complex situations, so they are willing to generate new ideas to return to their normal status [18].

Innovative behavior refers to the production, promotion, and realization of novel ideas in an organization, resulting in increased productivity and business performance. Novel ideas are required for making important changes in institutions, such as the creation of unique methods, the simplification of work procedures, and the use of new work tools [5]. Innovative behavior will give the organization a competitive advantage and ensure sustainability [6]. Employees’ innovative behavior leads to the growth and success of institutions [13].

In Egypt, all studies regarding PsyCap were descriptive [19]. At the international level, many studies have applied different models of PsyCap, but few of them have assessed the effect of PsyCap models on innovative behavior. Most of these international studies have assessed the effect of PPI and PCI models on performance, well-being, stress, motivation, and engagement. Additionally, there are limited studies on PsyCap among staff nurses, and most of them have focused on employees [15–17].

### Aim of the study

**Fig. 1:**
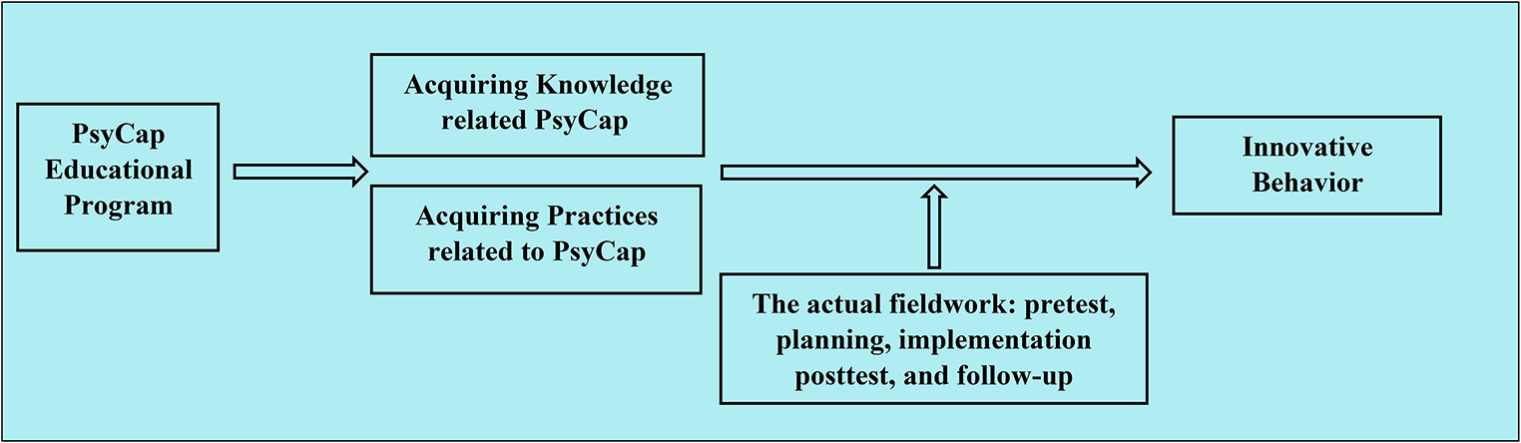
Conceptual model of the study

The present study aims to assess the effect of a PsyCap educational program on nurse interns’ innovative behavior. The study hypothesizes that a PsyCap educational program will improve nurse interns’ innovative behavior (Fig. 1).

## Methods

### Study design

A quasi-experimental design (a pretest – posttest one group) was used in this study.

### Study setting

This study was executed at Fayoum University hospitals affiliated with the Egyptian Ministry of Higher Education, where nurse interns completed the internship program at the units of these hospitals by rotation. Fayoum University hospitals in Fayoum city/Egypt include three hospitals: the surgical hospital specializes in providing surgical services for patients and has a capacity of 211 beds; the medical hospital specializes in providing medical services for patients and has a capacity of 160 beds; and the pediatric hospital specializes in providing medical and surgical services for pediatric patients and has a capacity of 104 beds.

### Study participants

The study participants comprised all the accessible and available nurse interns (*n* = 223) registered in the internship year (2022–2023) in the aforementioned settings during the data collection time. The whole number of nursing interns registered in the internship year (2022–2023) at Fayoum University hospitals was 240, but 17 of them were unavailable during the data collection time because of unexcused and excused absences. Neither inclusion criteria nor exclusion criteria were set because all available nurse interns were included. The study was carried out using a convenient sampling technique.

### Data collection instruments

The data were gathered using three instruments, namely, the Psychological Capital Knowledge Questionnaire, the Psychological Capital Questionnaire, and the Innovative Behavior Inventory.

#### Tool (I): Psychological capital knowledge questionnaire

The instrument was built by the authors, guided by a review of relevant literature [7, 16, 20], to assess the nurse interns’ knowledge regarding PsyCap throughout the program phases. It consists of two parts:

##### Part 1

This part focused on collecting personal data from the respondents, such as age, gender, marital status, level of income, having relatives in the nursing field, and pre-university education …. etc.

**Part 2**: This part consists of 53 multiple-choice questions. It was divided into three main dimensions regarding different areas of PsyCap: the theoretical framework of PsyCap (17 questions), the constructs of PsyCap (23 questions), and the models and approaches of PsyCap development (13 questions). The nurse interns’ responses were examined using a model key answer set by the authors. For each question, a score of “one” was given for the right response; in addition, “zero” was given for the false response. For each dimension of knowledge and total knowledge, the scores of the answers were summed, and the resulting score was divided by the number of questions, giving a mean score for each dimension of knowledge and total knowledge.

#### Tool (II): Psychological capital questionnaire

The instrument was developed by [20] and modified by the authors to assess the nurse interns’ perceptions of PsyCap. It consists of twenty-four items classified into four subscales: self-efficacy, hope, resilience, and optimism, and each subscale contains six items. The subjects’ responses were measured on a five-point Likert scale, ranging from “strongly disagree” with a score of “one” to “strongly agree” with a score of “five.” For each subscale of PsyCap and total PsyCap, the scores of the items were summed, and the resulting score was divided by the number of items, giving a mean score for each subscale of PsyCap and total PsyCap [21].

#### Tool (III): Innovative behavior inventory

The instrument was developed by [22] and modified by the authors to assess the nurse interns’ perceptions of innovative behavior. It consists of twenty-three items classified into seven main dimensions: idea generation (3 items), idea search (3 items), idea communication (4 items), implementation starting activities (3 items), involving others (3 items), overcoming obstacles (4 items), and innovation outputs (3 items). The subjects’ responses were measured on a five-point Likert scale, ranging from “never” with a score of “one” to “always” with a score of “five.” For each dimension of innovative behavior and total innovative behavior, the scores of the items were summed, and the resulting score was divided by the number of items, giving a mean score for each dimension of innovative behavior and total innovative behavior [23].

### Instruments validity and reliability

First, the authors developed and modified the study instruments based on a review of the literature, and then they were translated into Arabic format. Later, a jury group evaluated the face and content validity of the instruments. This group included nine specialists in the nursing administration and the mental health nursing from Cairo University, Ain Shams University, Fayoum University, Mansoura University, Damanhur University, and Suez Canal University in Egypt. The face and content validity sheet involved two parts: face validity included the opinions of the specialists about general appearance, format, and understanding of the instruments and accuracy of language, while content validity covered the opinions of specialists about the importance, applicability, and relevance of items. According to their opinions, the necessary modifications were made. Additionally, the instruments were examined for internal consistency (reliability) by employing Cronbach’s alpha coefficient. The Psychological Capital Knowledge Questionnaire had good reliability (0.74), while both the Psychological Capital Questionnaire and the Innovative Behavior Inventory had excellent reliability (0.918 and 0.922, respectively). As well, item analysis was performed for the Psychological Capital Knowledge Questionnaire. The difficulty index of the questions ranged from 0.33 to 0.74, and this indicates that the questions had average levels of difficulty. On the other hand, the discrimination index of the questions ranged from 0.23 to 0.39, and this indicates that the questions had good levels of discrimination. Finally, the authors performed a confirmatory factor analysis for the two adapted instruments to assess the relationship between the items and their dimensions, and the results indicate that both models had a good fit, as shown in Table 1. This preparatory phase lasted from mid-July 2022 until mid-December 2022.

**Table 1:** Results of the confirmatory factor analysis for the adapted instruments

	**Factors**	Self-efficacy		Hope		Resilience		Optimism
**Psychological Capital Questionnaire**	**Factor loadings**	They ranged from 0.62 to 0.85		They ranged from 0.61 to 0.84		They ranged from 0.63 to 0.79		They ranged from 0.62 to 0.78
	**Fit indices**	Root-Mean-Square Error of Approximation (RMSEA) = 0.044, Comparative Fit Index (CFI) = 0.963, Tucker-Lewis index (TLI) = 0.955, Goodness of Fit Index (GFI) = 0.0962, and χ2 = 959.47, DF = 424						
**Innovative Behavior Inventory**	**Factors**	Idea generation	Idea search	Idea communication	Implementing starting activities	Involving others	Overcoming obstacles	Innovation outputs
	**Factor loadings**	They ranged from 0.68 to 0.83	They ranged from 0.66 to 0.81	They ranged from 0.65 to 0.70	They ranged from 0.79 to 0.88	They ranged from 0.69 to 0.82	They ranged from 0.72 to 0.77	They ranged from 0.62 to 0.86
	**Fit indices**	Root-Mean-Square Error of Approximation (RMSEA) = 0.042, Comparative Fit Index (CFI) = 0.971, Tucker-Lewis index (TLI) = 0.966, Goodness of Fit Index (GFI) = 0.0968, and χ2 = 1042.02, DF = 444						

### Pilot study

Prior to gathering the data, the pilot study was executed with twenty-one nurse interns, representing 10% of the study participants. In addition to determining potential roadblocks and issues during the period of data collection, the pilot study sought to ascertain the instruments’ clarity, viability, and applicability, as well as the duration required by respondents to complete the questionnaires. No modification was made after the analysis of the sheets answered by the nurse interns, so the pilot sample was added to the main sample. This stage lasted from mid-December 2022 to its end.

### Ethical considerations

The Research Ethics Committee (REC) of the Faculty of Nursing/Ain Shams University in Cairo city/Egypt gave its approval to this study (code number 23.06.83) based on the standards of the committee and adhered to the Declaration of Helsinki. The Dean of the Faculty of Nursing/Ain Shams University sent official letters to the medical and nursing managers of Fayoum University hospitals in Fayoum city/Egypt, requesting their approval and collaboration for executing the study and gathering the data. These letters clarified the study’s purpose and its procedures. Informed written consent was received from each nursing intern after explaining all the study phases and being instructed about his or her right to leave the study without giving rationales. The nurse interns were guaranteed the anonymity and confidentiality of the data gathered.

### Fieldwork

The actual fieldwork for this study continued from the beginning of January 2023 until the end of November 2023; it took eleven months. The following five phases were used to implement the program:

**Phase I (Assessment)**: This phase lasted one month, from the beginning of January 2023 until its end. After receiving formal approvals for executing the study, the researcher met with the nursing managers of each hospital in order to schedule the most effective period for the data collection and program implementation. Then, the researcher met with all the nurse interns to clarify the aim and nature of the study and get written consent from them to take part. Then, the researcher distributed the instruments to all the nurse interns in order to assess their knowledge regarding PsyCap, their perceptions of PsyCap, and their perceptions of innovative behavior before implementing the PsyCap educational program. Later, the study instruments were personally collected and coded by the researcher. The collected data were considered baseline or pretest data, so they were analyzed by the researcher to assess nurse interns’ needs related to PsyCap. Each nurse intern took 30–35 min to complete the instruments.

#### Phase II (Planning)

This phase lasted one month, from the beginning of February 2023 until its end. Based on the results obtained from the data analysis in the assessment phase and a review of related literature, the researcher designed the PsyCap program for the nurse interns. Through the planning phase, the general aim of the program was set. The program aimed to improve the nurse interns’ knowledge and practices regarding PsyCap, as shown in Fig. 1. Additionally, the program plan was established, methods of teaching and media were detected. As well, the settings for holding the program sessions were reserved and equipped. As well, the program content, which covered the theoretical and practical aspects of PsyCap, was formulated, as shown in Table 2.

**Table 2:** The PsyCap educational program content

No	Sessions	Content
1	Capital	Opening the program, concept of the capital, and types of the capital
2	Mental health and positive psychology	Concept of mental health, objectives of mental health, factors affecting mental health, strategies for maintaining positive mental health, concept of positive psychology, general goals of positive psychology, and levels and topics of positive psychology
3	POB	Concept of POB, POB and positive organizational scholarship as shapes of positive psychology, criteria of POB, and psychological capacities meeting criteria of POB
4	Fundamentals of PsyCap	Concept of PsyCap, importance of PsyCap, components of PsyCap, criteria of PsyCap, factors affecting PsyCap, and theories of PsyCap
5	Effects of PsyCap	Stress and burnout, turnover intention, job search behavior, job satisfaction, motivation, customer services, organizational commitment, work engagement, organizational citizenship behavior, employee performance, and productivity and revenues
6	Self-efficacy	Concept of self-efficacy, criteria of self-efficacy, characteristics of self-efficacious people, constituents of self-efficacy, potential pitfalls of self-efficacy, and strategies strengthening self-efficacy
7	Hope	Concept of hope, importance of hope, components of hope, characteristics of hopeful managers and employees, causes of hopelessness, potential pitfalls of hope, and strategies developing hope
8	Resilience	Concept of resilience, importance of resilience, contents of resilience, characteristics of resilient managers and employees, factors affecting resilience, potential pitfalls of resilience, and strategies developing resilience
9	Optimism	Concept of optimism, importance of optimism, characteristics of optimists and pessimists, thinking styles of optimists and pessimists, potential pitfalls of optimism, and strategies developing optimism
10	Potential constructs of PsyCap	Concept of potential constructs of PsyCap, emotional intelligence, wisdom, humor, creativity, and well-being
11	Measurement of PsyCap and PPIs model	Concept of measuring PsyCap, characteristics of PsyCap measures, concept of PPIs model, importance of PPIs, features of PPIs model, and approaches to PPIs model
12	PCI Model	Concept of PCI model, features of PCI model, and format of PCI model (practices and activities strengthening self-efficacy, practices and activities developing hope, practices and activities developing resilience, and practices and activities developing optimism
13	RET approach	Concept of RET, goals and importance of RET, principles of RET, features of RET, guidelines to practice RET, and application of Albert Ellis’s model
14	Positive focus approach and SOAR (strengths, opportunities, aspirations, and results) framework	Concept of positive focus, benefits of positive focus, practices and activities developing positive focus, concept of SOAR framework as a personal branding, importance of SOAR framework, and building of the personal branding
15	PERMA model	Concept of PERMA model, general goal of PERMA model, uses of PERMA model, elements of PERMA model, effects of PERMA model, and application of PERMA model
16	Job crafting and career development approaches	Concept of job crafting, importance of job crafting, phases of job crafting, concept of career development, benefits of career development, and process of career development
17	Stress management approach	Concept of stress & stress management, types of stress, benefits of stress management, strategies of stress management, and ending the program

#### Phase III (Implementation)

This phase lasted four months, from the commencement of March 2023 until the last day of June 2023. During the implementation phase, the researcher divided the nurse interns into four groups because of their varied work schedules and large numbers. Every week, one session was presented to the four groups in a separate way (at different times and places). Each session lasted for two hours, from 12 p.m. to 2 p.m. (if presented during the day shifts) and from 10 p.m. to 12 a.m. (if presented during the night shifts). Based on the planning phase, the content of the program was presented through 17 sessions. Thus, the time allowed for the program was 34 h (20 theoretical hours and 14 practical hours).

At the commencement of the 1st session of the program, an orientation to the PsyCap program, the aims of the program, the importance of the program, and the content of the program was provided by the researcher. Before starting each session, an introduction to this session and its objectives was presented, and a review of the last session was provided. The program was implemented using various learning strategies. Lectures, brainstorming, and group work activities were part of these strategies. Additionally, educational media, such as data shows, whiteboards, and videos, were used. Through the implementation, different evaluation methods were used, such as a pretest and posttest to assess the effect of the session, verbal and nonverbal feedback for temporal assessment, and homework. The researcher distributed the handout of the program to be used as a memorial reference for nurse interns.

#### Phase IV (Post program evaluation)

This phase lasted one month through July 2023. The effects of the program on the nurse interns’ knowledge regarding PsyCap, perceptions of PsyCap, and perceptions of innovative behavior were immediately evaluated after the end of the program through the posttest. The posttest was implemented by using the same instruments as before. In this phase, the data collection procedure was applied in the same way as in the assessment phase.

#### Phase V (Follow-up)

This phase lasted one month through November 2023. By employing the same previous instruments, the follow-up test was conducted three months after the evaluation phase to examine the program’s impact again. In this phase, the data collection procedure was applied in the same way as in the assessment phase and post program evaluation phase.

### Statistical analysis

The Statistical Package for Social Science (SPSS) for Windows version 25.0 was used to enter, organize, and analyze the obtained data (IBM Corp., Armonk, NY, USA). The obtained data are displayed by employing descriptive statistics: frequencies and percentages for categorical data as well as means and standard deviations for quantitative data. A repeated measures ANOVA (F) was utilized to compare the means of quantitative variables among the three program phases (pretest, posttest, and follow-up). After employing the repeated measures ANOVA test, the post hoc test (Turkey) was used for two-by-two comparisons. Cronbach’s alpha coefficient was employed to examine the study instruments’ reliability. As well, item analysis was performed for the developed questionnaire to evaluate the questions’ difficulty and discrimination. Finally, confirmatory factor analysis was used for the adapted instruments to assess the relationship between the items and their dimensions. Differences were considered statistically significant for all the statistical tests if the p value was ≤ 0.05.

## Results

Table 3 shows that the nurse interns ranged in age from 22 to 25 years (23.23 ± 1.10 years), more than two-thirds (69.5%) were female, approximately three-quarters (74%) were single, more than half (52.5%) were living in rural areas, more than half (54.3%) had an average income, more than half (57%) had no hobbies, more than half (52.5%) had no relatives in the nursing field, less than two-thirds (62.3%) of the nurse interns registered in the college after general secondary education, less than two-thirds (61.4%) had entered college based on their academic score, and more than half (56.1%) had not previously worked as a health care provider while studying.

**Table 3:** Frequency distribution of the nurse interns’ personal characteristics (*n* = 223)

Personal characteristics	No.	%
**Age**: 22 years	25	11.2
23 years	127	57.0
24 years	56	25.1
25 years	15	6.7
Mean ± SD	23.23 ± 1.10	
**Gender**: Male	68	30.5
Female	155	69.5
**Marital status**: Single	165	74.0
Married	58	26.0
**Residence place**: Rural	117	52.5
Urban	106	47.5
**Level of income**: Low	98	43.9
Average	121	54.3
High	4	1.8
**Hobbies**: Yes	96	43.0
No	127	57.0
**Presence of relatives in nursing field**: Yes	106	47.5
No	117	52.5
**Pre-university education** General secondary education	139	62.3
Technical nursing institute	84	37.7
**Entry to the college based on**: Desire	86	38.6
Academic score	137	61.4
**Have you previously worked as a health care provider while studying?** Yes	98	43.9
No	125	56.1

Table 4 reveals that there were statistically significant improvements in the nurse interns’ mean scores regarding all dimensions of knowledge about PsyCap throughout both the posttest phase and the follow-up phase in comparison with the pretest phase, with p values ≤ 0.05. In general, there was a statistically significant improvement in the nurse interns’ mean scores regarding total knowledge about PsyCap throughout both the posttest phase and the follow-up phase in comparison with the pretest phase at p value ≤ 0.05.

**Table 4:** Comparison of the nurse interns’ knowledge regarding PsyCap throughout phases of the program (*n* = 223)

Knowledge dimensions	Pretest		Posttest		Follow-up		Repeated measures ANOVA		Post-hoc test (Turkey)		
									Pre-Post	Pre-Follow	Post-Follow
	Mean	SD	Mean	SD	Mean	SD	F	*p*	Mean difference (*p*)	Mean difference (*p*)	Mean difference (*p*)
Theoretical framework of PsyCap	3.61	1.06	13.25	3.13	12.43	2.83	301.69	< 0.001*	-9.64 (< 0.001*)	-8.82 (< 0.001*)	0.82 (0.79)
Constructs of PsyCap	5.57	1.87	18.08	4.05	16.56	4.01	287.72	< 0.001*	-12.51 (< 0.001*)	-10.99 (< 0.001*)	1.52 (0.48)
Models and approaches to PsyCap development	4.76	1.23	9.95	2.07	8.83	1.96	331.95	< 0.001*	-5.19 (< 0.001*)	-4.07 (< 0.001*)	1.12 (0.54)
**Total knowledge regarding PsyCap**	14.39	5.83	41.27	9.31	37.83	8.83	369.19	< 0.001*	-26.88 (< 0.001*)	-23.44 (< 0.001*)	3.44 (0.58)

(*) Statistically significant at *p* ≤ 0.05

Table 5 demonstrates that there were statistically significant improvements in the nurse interns’ mean scores regarding all the subscales of PsyCap as perceived by them throughout both the posttest phase and the follow-up phase in comparison with the pretest phase, with p values ≤ 0.05. In general, there was a statistically significant improvement in the nurse interns’ mean scores regarding total perception of PsyCap throughout both the posttest phase and the follow-up phase in comparison with the pretest phase at p value ≤ 0.05.

**Table 5:** Comparison of the nurse interns’ perceptions of PsyCap throughout phases of the program (*n* = 223)

PsyCap subscales	Pretest		Posttest		Follow-up		Repeated measures ANOVA		Post-hoc test (Turkey)		
									Pre-Post	Pre-Follow	Post-Follow
	Mean	SD	Mean	SD	Mean	SD	F	*p*	Mean difference (*p*)	Mean difference (*p*)	Mean difference (*p*)
Self-efficacy	17.35	2.50	23.11	3.43	22.08	3.36	85.44	< 0.001*	-5.76 (< 0.001*)	-4.73 (< 0.001*)	1.03 (0.51)
Hope	17.85	2.43	23.02	3.48	22.01	3.38	98.51	< 0.001*	-5.17 (< 0.001*)	-4.16 (0.002*)	1.01 (0.64)
Resilience	17.25	2.63	23.05	3.44	22.12	3.35	95.52	< 0.001*	-5.8 (< 0.001*)	-4.87 (< 0.001*)	0.93 (0.79)
Optimism	17.18	2.72	23.32	3.35	22.06	3.32	137.21	< 0.001*	-6.14 (< 0.001*)	-4.88 (< 0.001*)	1.26 (0.38)
**Total perception of PsyCap**	69.04	8.13	92.22	6.26	89.96	6.31	94.98	< 0.001*	-23.18 (< 0.001*)	-20.92 (< 0.001*)	2.26 (0.42)

(*) Statistically significant at *p* ≤ 0.05

Table 6 demonstrates that there were statistically significant improvements in the nurse interns’ mean scores regarding all the dimensions of innovative behavior as perceived by them throughout both the posttest phase and follow-up phase in comparison with the pretest phase, with p values ≤ 0.05. In general, there was a statistically significant improvement in the nurse interns’ mean scores regarding total perception of innovative behavior throughout both the posttest phase and the follow-up phase in comparison with the pretest phase at p value ≤ 0.05.

**Table 6:** Comparison of the nurse interns’ perceptions of innovative behavior throughout phases of the program (*n* = 223)

Innovative behavior dimensions	Pretest		Posttest		Follow-up		Repeated measures ANOVA		Post-hoc test (Turkey)		
									Pre-Post	Pre-Follow	Post-Follow
	Mean	SD	Mean	SD	Mean	SD	F	*p*	Mean difference (*p*)	Mean difference (*p*)	Mean difference (*p*)
Idea generation	8.71	0.31	11.85	0.43	11.02	0.32	109.82	< 0.001*	-3.14 (< 0.001*)	-2.31 (0.002*)	0.83 (0.94)
Idea search	8.87	0.19	11.63	0.57	11.18	0.20	94.28	< 0.001*	-2.76 (< 0.001*)	-2.31 (0.005*)	0. 45 (1.04)
Idea communication	10.53	0.82	15.10	1.14	14.18	1.09	119.35	< 0.001*	-4.57 (< 0.001*)	-3.65 (< 0.001*)	0.92 (0.56)
Implementing starting activities	7.26	0.65	11.21	1.07	10.92	0.74	171.65	< 0.001*	-3.95 (< 0.001*)	-3.66 (< 0.001*)	0.29 (1.18)
Involving others	7.78	0.43	11.25	1.03	11.02	0.29	123.58	< 0.001*	-3.47 (< 0.001*)	-3.24 (< 0.001*)	0.23 (1.95)
Overcoming obstacles	10.17	0.88	15.15	1.09	14.09	1.14	131.38	< 0.001*	-4.98 (< 0.001*)	-3. 92 (< 0.001*)	1.06 (0.19)
Innovation outputs	7.74	0.59	11.12	1.11	10.84	0.81	121.12	< 0.001*	-3.38 (< 0.001*)	-3.1 (< 0.001*)	0.28 (1.24)
**Total perception of innovative behavior**	60.55	7.15	91.31	9.06	88.89	8.33	141.77	< 0.001*	-30. 76 (< 0.001*)	-28.34 (< 0.001*)	2.24 (0.59)

(*) Statistically significant at *p* ≤ 0.05

## Discussion

High levels of PsyCap increase nurses’ confidence and stimulate positive thinking. This increases their job satisfaction and organizational commitment. Additionally, PsyCap has a positive impact on nurses’ engagement and intrinsic motivation [13]. Nurses’ innovative behaviors are imperative assets that make hospitals successful. Nursing innovation not only develops the nursing industry but also improves the quality of care and patient prognosis [18]. Thus, this study aimed to assess the effect of a PsyCap educational program on nurse interns’ innovative behavior.

**Regarding the nurse interns**’ **knowledge regarding PsyCap**, this study showed that there were statistically significant improvements in all the dimensions of knowledge and total knowledge regarding PsyCap among the nurse interns throughout both the posttest phase and the follow-up phase in comparison with the pretest phase. These statistically significant improvements were attributed to the successful implementation of the PsyCap program because it was first implemented according to the interns’ pre-assessed results; second, information was well presented by suitable educational aids; and third, the program booklet presented to them was rich in theoretical knowledge about PsyCap. Additionally, the nurse interns were highly ready to acquire new knowledge from the PsyCap program. The current findings are consistent with those of a study conducted by Kalman and Summak [24] in Turkey to evaluate the effects of PsyCap development training, and the findings showed that subjects’ knowledge about PsyCap significantly increased after implementing the training. Another study was executed by Zhang et al. [25] in China to examine the effectiveness of the PsyCap intervention (PCI) program on employees’ behaviors, and the findings showed that the reading material presented to the intervention group significantly improved their knowledge about PsyCap at the posttest time.

**Regarding the nurse interns**’ **perceptions of PsyCap**, this study revealed that there were statistically significant improvements in all the components of PsyCap and total PsyCap as perceived by the nurse interns throughout both the posttest phase and the follow-up phase in comparison with the pretest phase. These statistically significant improvements were attributed to the successful implementation of the PsyCap program, which aimed to improve the nurse interns’ knowledge and practices regarding PsyCap. The program provided the nurse interns with models and approaches to the development of their PsyCap perceptions, such as the PCI model, the PPI model, the PERMA model, and the SOAR framework, and the program booklet and interactive worksheets were rich in applications related to PsyCap. The current findings are consistent with those of a study conducted by Chaleoykitti and Thaiudom [26] in Thailand to investigate the effect of the PsyCap program on the retention of nurses, and the findings showed that the levels of nurses’ PsyCap in the experimental group significantly increased after the PsyCap program. Additionally, a meta-analysis study was conducted by Lupșa et al. [9] in Romania to assess the overall impact of interventions prepared to promote employees’ PsyCap, and the findings showed that their effects on self-efficacy, hope, resilience, and optimism were significant through the posttest and follow-up phases. Conversely, the current findings disagree with those of a study conducted by Hodges [27] in the USA to explore the impact of PsyCap micro-intervention on employees’ performance and engagement, and the findings showed that the micro-intervention had no significant effect on PsyCap in the posttest phase.

**Regarding the nurse interns**’ **perceptions of innovative behavior**, this study demonstrated that there were statistically significant improvements in all the dimensions of innovative behavior and total innovative behavior as perceived by the nurse interns throughout the posttest phase and follow-up phase in comparison with the pretest phase. These statistically significant improvements were attributed to the positive effects of successfully implementing the PsyCap program because it first made the nurse interns motivated by new challenges and risk-taking behavior related to the practice of innovative behavior and confident in their abilities to apply innovative practices at hospitals; second, it made them resilient in dealing with work environment difficulties that hindered innovative behavior; third, it made them optimistic, and when optimists were faced with negative outcomes, they were likely to attribute the failure to external causes, thereby avoiding a reduction in the effort of innovative practices; and fourth, it made them hopeful and persistent to practice innovative behavior for achieving their goals with full desire. The current findings are consistent with those of a study conducted by Costantini et al. [28] in Italy to improve employees’ behaviors by applying a PsyCap intervention program, and the findings showed that there was a statistically significant improvement in creativity performance throughout the posttest phase. Additionally, a study was executed by Ibrahim et al. [29] in Egypt to assess the effectiveness of a self-efficacy program on nurses’ innovative behaviors, and the findings showed that self-efficacy training as a part of the PsyCap program significantly improved all the dimensions of innovative behavior of nurses in the posttest phase. These improvements were slightly reduced in the follow-up phase.

### Limitations

Despite the positive results, there are limitations to consider. There is a limitation related to the shortage of previous research studies focused on assessing the effect of PsyCap interventions on innovative behavior. Thus, there is a strong need for more studies on this topic to foster and generalize the current findings. Another limitation is related to the models and approaches used to develop PsyCap. This study applied two models together; however, all previous studies focused on the application of only one model. Thus, it’s difficult to separate the effects of the two models from each other. Additionally, the study’s focus on newly graduated nurses may limit the universality of the results to experienced nurses. Finally, the data were self-reported, which could limit the generalizability of the study results because of the risk of bias.

## Conclusion

There were statistically significant improvements in nurse interns’ knowledge regarding PsyCap, perceptions of PsyCap, and perceptions of innovative behavior throughout both the posttest phase and the follow-up phase in comparison with the pretest phase. In light of these findings, it was concluded that the psychological capital educational program was effective and beneficial in improving the nurse interns’ perceptions of innovative behavior, so the research hypothesis was accepted.

### Implications for practice

The study findings are beneficial for the application of these PsyCap interventions in hospitals through professional development programs and orientation programs presented to healthcare providers to improve their psychological well-being and innovative behavior, and this will reflect on the quality of patient care and organizational success. Additionally, the booklet of the current PsyCap program is recommended for use as a reading handout by healthcare providers in healthcare settings. Quality assurance units in hospitals should publish posters containing tips for improving PsyCap. On the other hand, the nursing curricula should be updated to include PsyCap in undergraduate courses. Additionally, the faculty of nursing should carry out periodic follow-ups through quarterly or semi-annual questionnaires on nurse interns’ perceptions of PsyCap during the internship program. Further research studies are suggested for replicating the same study in different settings and with different populations. As well, other studies are required to investigate the effects of the PsyCap program for healthcare providers on the quality of patient care.

## Data Availability

Due to confidentiality concerns, public access to the materials and data utilized in this study is not permitted. On reasonable request, they can be obtained from the corresponding author.

## Abbreviations

PsyCap: Psychological capital
POB: Positive organizational behavior
PCI: Psychological capital intervention
PPIs: Positive psychology interventions
PERMA: Positive emotion, engagement, relationships, meaning, and accomplishment
SOAR: Strengths, opportunities, aspirations, and results
RET: Rational-emotive therapy
